# Combined Effect of *CYP2C19* Genetic Polymorphisms and C-Reactive Protein on Voriconazole Exposure and Dosing in Immunocompromised Children

**DOI:** 10.3389/fped.2022.846411

**Published:** 2022-03-21

**Authors:** Juan Chen, Ying Wu, Yuelin He, Xiaoqin Feng, Yuqiong Ren, Shiting Liu

**Affiliations:** ^1^Department of Pharmacy, Nanfang Hospital, Southern Medical University, Guangzhou, China; ^2^Department of Biostatistics, Guangdong Provincial Key Laboratory of Tropical Disease Research, School of Public Health, Southern Medical University, Guangzhou, China; ^3^Department of Pediatrics, Nanfang Hospital, Southern Medical University, Guangzhou, China

**Keywords:** children, voriconazole, trough concentration, dose requirements, therapeutic drug monitoring, *CYP2C19*, genetic polymorphism, C-reactive protein

## Abstract

**Background:**

Pediatric patients have significant interindividual variability in voriconazole exposure. The aim of the study was to identify factors associated with voriconazole concentrations and dose requirements to achieve therapeutic concentrations in pediatric patients.

**Methods:**

Medical records of pediatric patients were retrospectively reviewed. Covariates associated with voriconazole plasma concentrations and dose requirements were adjusted by using generalized linear mixed-effect models.

**Results:**

A total of 682 voriconazole steady-state trough concentrations from 91 Chinese pediatric patients were included. Voriconazole exposure was lower in the *CYP2C19* normal metabolizer (NM) group compared with the intermediate metabolizer (IM) group and the poor metabolizer (PM) group (*p* = 0.0016, *p* < 0.0001). The median daily dose of voriconazole required to achieve therapeutic range demonstrated a significant phenotypic dose effect: 20.8 mg/kg (range, 16.2–26.8 mg/kg) for the *CYP2C19* NM group, 18.2 mg/kg (range, 13.3–21.8 mg/kg) for the *CYP2C19* IM group, and 15.2 mg/kg (range, 10.7–19.1 mg/kg) for the *CYP2C19* PM group, respectively. The extent of impact of C-reactive protein (CRP) levels on voriconazole trough concentrations and dose requirements varied between *CYP2C19* phenotypes. Increases of 20, 120, 245, and 395 mg/L from 5 mg/L in CRP levels were associated with increases in voriconazole trough concentration by 22.22, 50, 64.81, and 75% respectively, in the NM group; by 39.26, 94.48, 123.93, and 146.63%, respectively, in the IM group; and by 17.17, 37.34, 46.78, and 53.65%, respectively, in the PM group. Meanwhile, increases of 20, 120, 245, and 395 mg/L from 5 mg/L in CRP levels were associated with increases in voriconazole dose requirements by 7.15, 14.23, 17.35, and 19.43%, respectively, in the PM group; with decreases in voriconazole dose requirements by 3.71, 7.38, 8.97, and 10.03%, respectively, in the NM group; and with decreases by 4, 9.10, 11.05, and 12.35%, respectively, in the IM group. In addition, age and presence of immunosuppressants had significant effects on voriconazole exposure.

**Conclusions:**

Our study suggests that *CYP2C19* phenotypes, CRP concentrations, age, and the presence of immunosuppressants were factors associated with the pharmacokinetic changes in voriconazole. There was heterogeneity in the effect of CRP on voriconazole plasma concentrations across different *CYP2C19* genotypes. Combining relevant factors with dose adaptation strategies in therapeutic drug monitoring may help to reduce the incidence of subtherapeutic and supratherapeutic concentrations in clinical practice.

## Introduction

Invasive fungal infections are significant causes of morbidity in immunocompromised pediatric patients with myelosuppressive chemotherapy or hematopoietic stem cell transplantation (HSCT) (1, 2). Voriconazole is a broad-spectrum antifungal agent used for invasive fungal infections prophylaxis and treatment (3, 4). Pediatric patients have larger interindividual and intraindividual variability compared with adults (5, 6); thus, therapeutic drug monitoring (TDM) is essential for optimizing voriconazole dosing regimens in pediatric patients. Whereas most of the published studies were focused on adult patients, there are few studies of voriconazole TDM and dosage regimens in pediatric patients, especially for the Asian population. The drug package leaflet recommends a weight-based dose of 9 mg/kg per 12 h intravenous (IV) or oral (PO) for 2- to 12-year-old children. The loading dose is 6 mg/kg per 12 h IV for patients older than 12 years. To our knowledge, there is rarely evidence on the optimal dose regimens for pediatric patients, although the pharmacokinetics of voriconazole in such a population may be influenced by more factors in different degrees or mechanisms compared with adults.

Voriconazole is metabolized by the cytochrome P450 enzymes, and CYP2C19 is the primary enzyme responsible for the metabolism of voriconazole. CYP2C19 is highly polymorphic. The majority of individuals will carry the ^*^1, ^*^2, ^*^3, or ^*^17 alleles. The activity of CYP2C19 enzyme demonstrates significant individual diversity. The wild-type CYP2C19^*^1 allele expresses a normal function CYP2C19 enzyme; CYP2C19^*^2 (c.681G > A; rs4244285) and CYP2C19^*^3 (c.636G > A; rs4986893) encode no function allele. In contrast, CYP2C19^*^17 allele (c.-806C > T; rs12248560) results in increased enzyme activity. Based on *CYP2C19* allelic function, patients could be divided into five phenotypes: ultrarapid metabolizer (UM), rapid metabolizer (RM), normal metabolizer (NM), intermediate metabolizer (IM), or poor metabolizer (PM) (7). The *CYP2C19* allele and phenotype frequencies are different between Caucasians or Africans and Asians (8, 9). The ethnic differences of *CYP2C19* genetic polymorphism imply different plasma concentration and dose requirement in Asian population. The impact of *CYP2C19* polymorphisms on voriconazole metabolism and variability in exposure in adults has been acknowledged (10–12). Limited and controversial statistical data are available describing the relationship between *CYP2C19* polymorphisms and voriconazole exposure [Caucasians (5, 6, 13–16), Asians (17, 18)] and dose requirement (13, 19, 20) in pediatric patients. Hence, whether the genotype-directed dosing allows for optimized voriconazole concentration in pediatric patients needs more studies.

Inflammations are frequently observed in patients with myelosuppressive chemotherapy or HSCT. The inflammation, reflected by C-reactive protein (CRP) concentrations, was associated with elevated voriconazole trough concentration in adults (21–23); the effect of inflammation on voriconazole concentrations in pediatric patients was rarely studied. A previous study demonstrated that the CRP value seems to be associated with higher voriconazole trough concentrations only in children ≥12 years old (24, 25). In the present study, the CRP levels in pediatric patients were hypothesized to impact voriconazole exposure in a *CYP2C19* phenotype-dependent way. Moreover, the pharmacokinetics of voriconazole were reported to be influenced by other factors including age, gender, route of administration, drug–drug interactions, and liver and renal function (26), whose effects have also been considered in the analysis in this study.

Therefore, a retrospective study was performed in immunocompromised pediatric patients who were treated with voriconazole. Sociodemographic, clinical, voriconazole TDM, dose adjustment, and *CYP2C19* genetic data were collected. The aim of the study was to describe the TDM and dose requirements of voriconazole, as well as to identify the determinants of the variability in voriconazole exposure and hence dose requirement to achieve the therapeutic range in Asian children.

## Materials and Methods

### Study Design and Patient Population

A single-center retrospective study of immunocompromised children <18 years of age who were treated with voriconazole as an inpatient over a 3-year period (May 2017-May 2020) at Department of Pediatric Hematology, Nanfang Hospital of Southern Medical University, China, was performed. Inclusion criteria were aged <18 years with routine TDM of voriconazole and taken as trough concentrations at steady state, which was defined as after 3 days of treatment (without loading dose) or dose adjustment (27, 28). Patients with potential interacting drugs used concomitantly (such as strong inhibitor or inducer of CYP450 described in the summary of product information), liver cirrhosis, chronic renal failure (with estimated creatine clearance ≤ 60 ml/min) were excluded. Individuals were genotyped for *CYP2C19* at the discretion of physicians. The following data were extracted from the clinical chart: (1) demographic characteristics, including gender, age, body weight, underlying disease; (2) voriconazole treatment and TDM data, including daily dosage (mg/kg), route of administration, and plasma trough concentrations; (3) other factors that could potentially influence the voriconazole trough concentration, laboratory parameters such as CRP; total protein (TP); albumin (ALB); total bilirubin (TBIL); direct bilirubin (DBIL); alanine aminotransferase (ALT); aspartate transaminase (AST); serum creatinine (CR), which were measured within the same day; and the concomitant medication. The concomitant medications were immunosuppressants used including cyclosporine, tacrolimus, and sirolimus; glucocorticoids used including prednisone, methylprednisolone, and dexamethasone; and proton pump inhibitors (PPIs) used including omeprazole and esomeprazole.

This study was conducted in accordance with the Declaration of Helsinki and was approved by the Institutional Review Board of Nanfang Hospital under reference number NFEC-2020-053.

### Genotyping and Phenotype Assignment

*CYP2C19* genotyping was performed by a validated fluorescence *in situ* hybridization method (Beijing Precision Medical Platform analysis software) (29). Blood samples (3 ml EDTA) were collected and pretreated (blood samples were collected before the allograft for the patients of HSCT). Samples were placed in fluorescent detector (Xi'an Tianlong Science and Technology Co., Ltd.). Different *CYP2C19* genotype fluorescence signal was automatically obtained by fluorescence *in situ* hybridization and chromosome karyotype analysis system. *CYP2C19* genotype was determined for the ^*^2, ^*^3, and ^*^17 alleles, the presence of the wild-type allele *CYP*2*C*19^*^1 was inferred in the absence of *CYP*2*C*19^*^2, *CYP*2*C*19^*^3, and *CYP*2*C*19^*^17, which define the major *CYP2C19* phenotypes. Based on *CYP2C19* genotypes in our study, phenotypes were divided into four categories: NM (*CYP*2*C*19^*^1/^*^1), IM (*CYP*2*C*19^*^1/^*^2, *CYP*2*C*19^*^2/^*^17 or *CYP*2*C*19^*^1/^*^3), or PM (*CYP*2*C*19^*^2/^*^2, *CYP*2*C*19^*^2/^*^3 or *CYP*2*C*19^*^3/^*^3) according to the Clinical Pharmacogenetics Implementation Consortium guidelines (7).

### Determination of Voriconazole Plasma Concentration

For each patient, the voriconazole trough concentrations were determined on blood sample withdraw within a 1-h period before the next administration. Plasma concentrations of voriconazole were measured by high-performance liquid chromatography–tandem mass spectrometry method. Sample preparation included protein precipitation by mixing 100 μl of plasma with 300 μl of acetonitrile containing 6,7-dimethyl-2, 3-di(2-pyridyl) quinoxaline (internal standard, 500 ng/ml). Two microliters of the supernatant was injected into the chromatographic system after centrifugation at 14,000 revolutions/min for 15 min. Separation conditions were the following: Agilent Poroshell 120 EC-C18 column (3.0 × 50 mm, 2.5-μm); mobile phase composed initially of 20:80 acetonitrile with formic acid (0.1%)/water with formic acid (0.1%) using isocratic elution as the mode of separation; flow rate of 0.5 ml/min; column temperature of 30°C. The plasma concentration of voriconazole was linear (*r* > 0.999) over the range of 0.1-10 μg/ml. The intraday and interday accuracy and precision data did not exceed 15% and could be used to determine voriconazole trough concentration accurately. The therapeutic range of voriconazole was between 1 and 5 μg/ml (30, 31). The CDR (concentration-to-dose ratio) value was applied to standardize voriconazole concentration.

### Statistical Analysis

The Hardy–Weinberg equilibrium (HWE) test was performed using an appropriate χ^2^ test, with *p* < 0.05 indicating a lack of agreement with HWE. To carry out the descriptive analysis, continuous and nonnormal variables were expressed as median and interquartile range. Categorical variables were summarized as frequency and percentage. Demographics (age, gender), clinical characteristics (route of administration, voriconazole dose, laboratory parameters reflected inflammation status, liver and kidney function, coadministered medications), and *CYP2C19* genotype were possible factors considered in our study. The crude correlation between CRP levels and voriconazole exposure was analyzed by the Spearman rank correlation test. A univariate linear mixed-effects regression analysis was conducted to identify covariates associated with voriconazole, and *p* = 0.15 was used as the entry threshold. Then, a multivariate generalized linear mixed-effects model was analyzed based on the results of univariate analyses to assess the effect of covariates on voriconazole concentration and dose requirement, with the patient effect specified as a random effect. Nonnormal quantitative variables were transformed by using their logarithmic value (voriconazole CDR, CRP, and weight-corrected dose). The following confounding factors were introduced into the final model for adjustment: age, gender, weight-corrected dose, presence of immunosuppressants, CRP, *CYP2C19* phenotypes, and the interaction term of CRP and phenotypes. A *p* < 0.05 was considered to be statistically significant, except for subgroup analyses where a *p*-value threshold of 0.017 was considered (adjustment for multiple comparisons). All statistical analyses were performed with SAS 9.3 (SAS Institute Inc., Cary, NC, USA). Graphic operations were completed by R Language (version 4.1.2).

## Results

### Population Characteristics

A total of 682 voriconazole steady-state trough concentrations from 91 Chinese pediatric patients were qualified for this study. The demographic characteristics, type of HSCT, underlying diseases, *CYP2C19* genotypes, laboratory parameters, and coadministered medication are summarized in Table 1. The majority was male (59.3%) and had a recent history of HSCT (75.8%). The main underlying disease in our study was β-thalassemia major followed by hematologic malignances. *CYP2C19* allele frequencies were 29.7% for ^*^2, 3.8% for ^*^3 and 0.5% for ^*^17. None of the genotypes deviated from HWE. There were 41 (45.1%), 40 (44.0%), and 10 (10.9%) patients with *CYP2C19* NM, IM, and PM, respectively. The mean concentration of CRP was 11 mg/L (range, 2.7–44.1 mg/L), which was above the upper limit of normal. The median levels of other laboratory parameters were inside the normal range; 64.5, 19.2, and 18.6% of voriconazole were used simultaneously with immunosuppressants, PPIs, and glucocorticoids, respectively.

**Table 1:** Demographic and clinical characteristics of the study subjects (*N* = 91).

**Characteristics**	**No. of patients (%) or median (interquartile range)**
Age (years)	6.0 (3.0–9.0)
**Gender**	
Male	54 (59.3%)
Female	37 (40.7%)
**Weight (kg)**	18.3 (13.5–23.9)
**HSCT**	69 (75.8%)
**Type of HSCT**	
PBSC	42 (60.9%)
PBSC-CB	27 (39.1%)
**Underlying disease**	
β-Thalassemia major	43 (47.2%)
Acute myeloid leukemia	26 (28.6%)
Acute lymphoblastic leukemia	5 (5.5%)
Severe aplastic anemia	6 (6.6%)
Juvenile myelomonocytic leukemia	3 (3.3%)
Adrenal neuroblastoma	2 (2.2%)
Myeloid sarcoma	1 (1.1%)
Myelodysplastic syndrome	1 (1.1%)
Albers–Schönberg disease	1 (1.1%)
Chronic active Epstein–Barr virus infection	1 (1.1%)
Wiskott–Aldrich syndrome	1 (1.1%)
Chronic granulomatous disease	1 (1.1%)
***CYP2C19*** **phenotypes**	
NM	41 (45.1%)
IM	40 (44.0%)
PM	10 (10.9%)
***CYP2C19*** **diplotypes**	
*1/*1	41 (45.1%)
*1/*2	36 (39.5%)
*1/*3	3 (3.3%)
*2/*17	1 (1.1%)
*2/*2	8 (8.8%)
*2/*3	1 (1.1%)
*3/*3	1 (1.1%)
**Laboratory parameters**	
CRP (mg/L)	11 (2.7–44.1)
TP (g/L)	64.8 (59.7–69.7)
ALB (g/L)	38.6 (35.2–41.5)
ALT (U/L)	16 (10–33)
AST (U/L)	22 (15–32)
TBIL (μmol/L)	8.6 (5.6–12.1)
DBIL (μmol/L)	3.3 (2.2–4.9)
CR (μmol/L)	25 (19–32)
**Coadministered medication**	
Immunosuppressants	440 (64.5%)
PPIs	131 (19.2%)
Glucocorticoids	127 (18.6%)

*CB, cord blood; PBSC, peripheral blood stem cell*.

### Voriconazole Therapy, Dose Adaptation, and TDM

A total of 682 voriconazole steady-state trough concentrations were measured, representing a median of six voriconazole concentrations per patient (range, 4–9 measurements) for a median follow-up duration of voriconazole therapy of 59 days (range, 23–149 days). The median of trough concentration was 1.7 μg/ml (range, 0.9–3.1 μg/ml). Thirty-eight percent of trough concentrations were out of the therapeutic range. The interpatient variability of trough concentrations was 105.63%, and the median intrapatient variability of trough concentrations was 62.2% (range, 49.1–84.3%). There were 281 (41.2%), 308 (45.2%), and 93 (13.6%) samples with *CYP2C19* NM, IM, and PM, respectively. The descriptions of voriconazole therapy, dose adaptation, and TDM of the three *CYP2C19* phenotype groups are displayed in Table 2. The median daily dose per weight required for achieving target concentrations was 18.4 mg/kg (range, 14.3–22.2 mg/kg). Voriconazole IV-to-PO switch was performed for sequential therapy before inpatients were discharged from the hospital. Frequency of voriconazole PO therapy was 26.8% in our study. During the course of treatment of 91 patients, a total of 281 dose adaptations were made, of which 179 had dose increases and 102 had dose decreases. One hundred eleven (62.0%) had dose increases when trough concentrations were <1 μg/ml, and 44 (43.1%) had dose decreases when trough concentrations were >5 μg/ml. After dose adjustments in cases out of the therapeutic range, 92 cases (59%) reached the therapeutic range.

**Table 2:** Voriconazole therapy, dose adaptation, and therapeutic drug monitoring.

	***n*** **(%) or median (interquartile range)**			
	**ALL**	***CYP2C19* NM**	***CYP2C19* IM**	***CYP2C19* PM**
**Therapeutic drug monitoring**				
TDM per patient n (%)	6 (4–9)	6 (4–8)	6 (4–10.7)	8.5 (5.7–13.5)
Voriconazole C_trough_	1.7 (0.9–3.1)	1.2 (0.6–2.3)	1.9 (1.1–3.2)	2.5 (1.6–4.2)
Level of C_trough_
<1 (μg/ml)	194 (28.4%)	120 (42.7%)	64 (20.8%)	10 (10.8%)
1–5 (μg/ml)	422 (61.9%)	145 (51.6%)	209 (67.8%)	68 (73.1%)
>5 (μg/ml)	66 (9.7%)	16 (5.7%)	35 (11.4%)	15 (16.1%)
**Voriconazole therapy**				
Daily dose/weight (mg/kg) for achieving therapeutic range	18.4 (14.3–22.2)	20.8 (16.2–26.8)	18.2 (13.3–21.8)	15.2 (10.7–19.1)
Intravenous (mg/kg)	18.6 (14.6–22.5)	20.7 (16–26.1)	18.3 (14.1–21.9)	15.4 (11.5–19.8)
Oral (mg/kg)	16.9 (13.5–22.1)	20.8 (16.1–27.3)	17.3 (11.9–21.3)	14 (10.3–15.7)
Oral therapy n (%)	183 (26.8%)	69 (24.6%)	86 (27.9%)	28 (30.1%)
Dose adaptations*				
Frequency of dose adaptations (%)	40 (25–50)	50 (29.2–57.9)	36 (25–50)	34.9 (24.5–43.2)
Frequency of dose increase (%)	25 (12.5–37.5)	33.3 (23.6–50)	21.8 (9.3–33.3)	14.6 (7.3–22.1)
Frequency of dose decrease (%)	12.5 (0–21.4)	0 (0–20)	13.4 (0–25)	17.4 (13.8–23.3)

^*^Frequency of dose adaptations: number of dose adaptation/number of C_trough_ measurements; Frequency of dose increase: number of dose increase adaptation/number of C_trough_ measurements; Frequency of dose decrease: number of dose decrease adaptation/ number of C_trough_ measurements.

### Influence of *CYP2C19* Genetic Polymorphism on Voriconazole Concentrations and Dose Requirements

**Figure 1:**
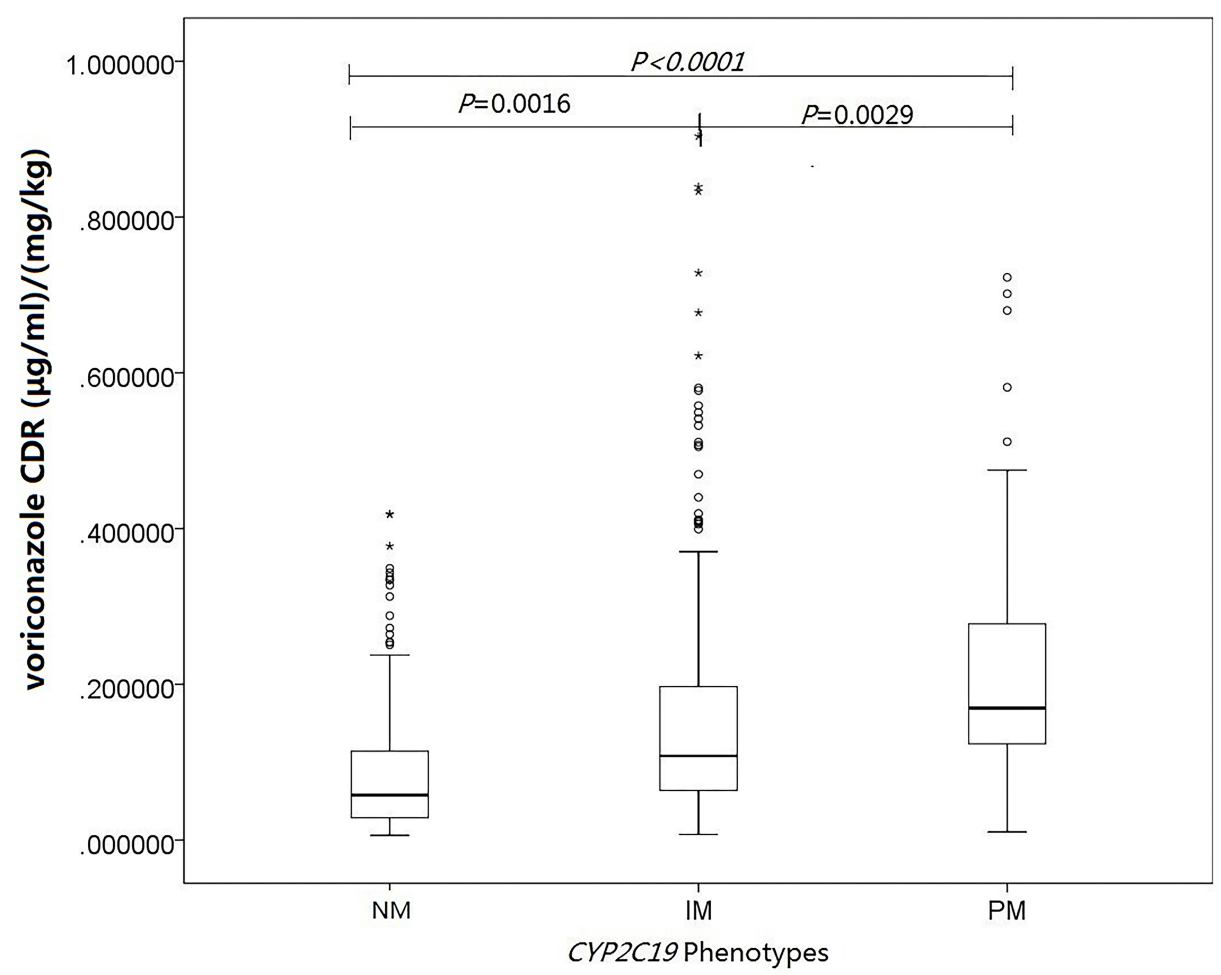
Influence of *CYP2C19* genotype and other covariates on voriconazole concentrations. Linear mixed-effects regression analysis of the relationship between *CYP2C19* phenotypes and voriconazole concentration-to-dose (μg/ml per mg/kg). The asterisks represent extreme outliers.

**Figure 2:**
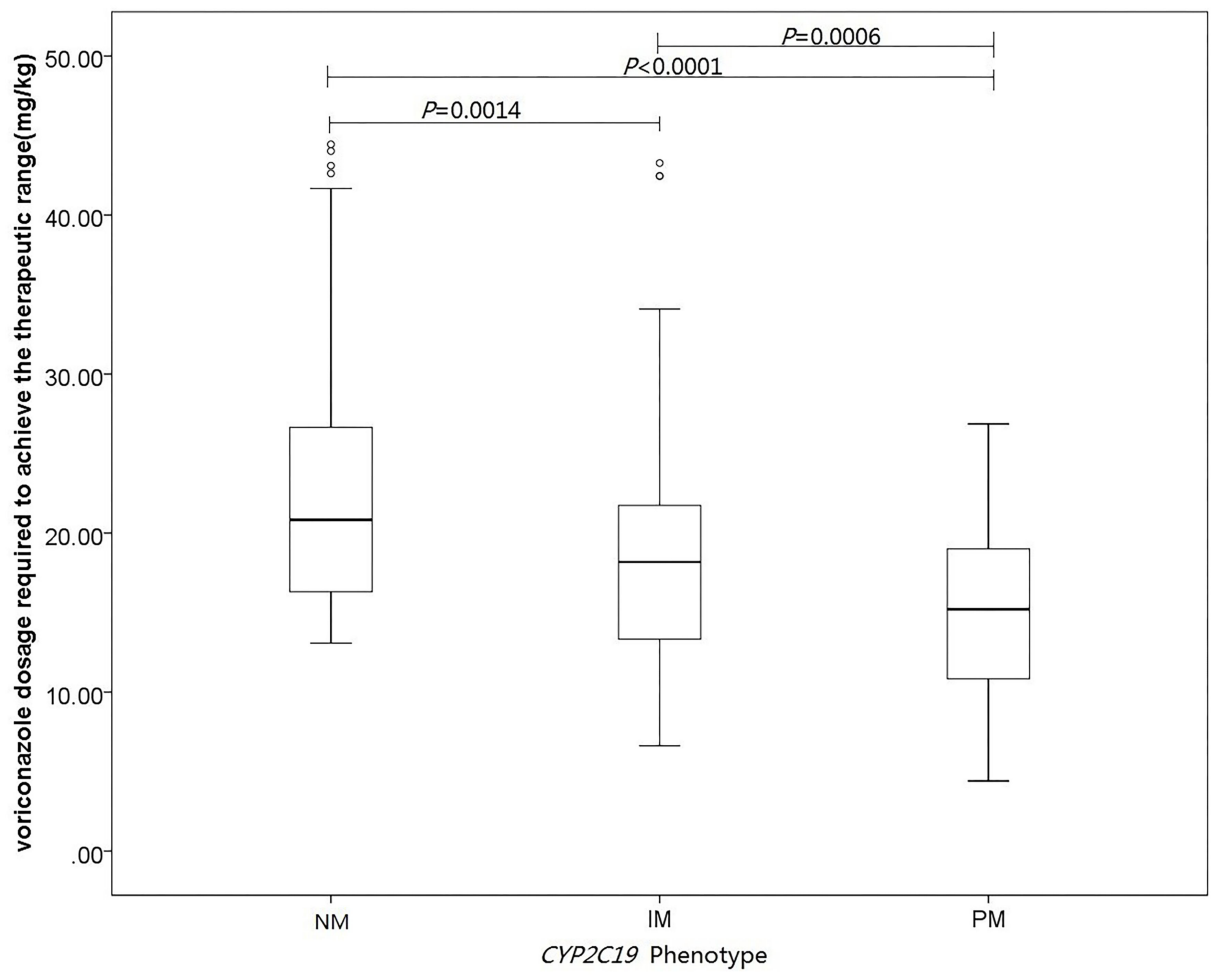
Influence of *CYP2C19* genotype and other covariates on voriconazole dose requirements. Linear mixed-effects regression analysis of the relationship between *CYP2C19* phenotypes and voriconazole dose required to achieve the therapeutic range (mg/kg).

The influence of *CYP2C19* genotype was explored on the voriconazole CDR to overcome the influence of voriconazole dose. The median levels of voriconazole CDR in the *CYP2C19* NM, IM, and PM groups were 0.0578 μg/ml per mg/kg (range, 0.0279–0.114 μg/ml per mg/kg), 0.108 μg/ml per mg/kg (range, 0.0639–0.197 μg/ml per mg/kg), and 0.169 μg/ml per mg/kg (range, 0.123–0.279μg/ml per mg/kg), respectively. In multiple linear mixed-effects regression model, *CYP2C19* phenotypes affected voriconazole concentration (Figure 1; Table 3.1), with a higher voriconazole CDR in the *CYP2C19* IM and PM vs. *CYP2C19* NM group (*p* = 0.0016, *p* < 0.0001), respectively. The median daily dose of voriconazole required to achieve therapeutic range was higher in the *CYP2C19* NM group and demonstrated an phenotypic dose effect: 20.8 mg/kg (range, 16.2–26.8 mg/kg) for the *CYP2C19* NM group compared with 18.2 mg/kg (range, 13.3–21.8 mg/kg) for the *CYP2C19* IM group and 15.2 mg/kg (range, 10.7–19.1 mg/kg) for the *CYP2C19* PM group (*p* = 0.0014, *p* < 0.0001), respectively (Figure 2; Table 3.2). The effects of *CYP2C19* diplotypes on voriconazole CDR and dose requirements are depicted in Supplementary Figures 1, 2. There were some visual trends between different *CYP2C19* diplotypes; the statistical analysis was not performed for limited sample size.

**Table 3.1:** Multivariate linear mixed-effects regression analysis of the influence of covariates on voriconazole CDR (μg/ml per mg/kg).

**Factor**	**Coefficient**	***P*-value**
Age	0.07127	**<0.0001**
**Gender**		
Male	—	—
Female	−0.1030	0.4426
**Phenotypes**		
NM	—	—
IM	0.5185	**0.0016**
PM	0.9491	**<0.0001**
**Presence of immunosuppressants**		
No	—	—
Yes	−0.2691	**0.0103**
Logarithmic of weight-corrected dose	0.1574	0.1085
Logarithmic of CRP	0.06425	**0.0397**
**Interactive effects of CRP and Phenotypes**		
CRP*NM	0.006769	**0.0001**
CRP*IM	0.004098	**<0.0001**
CRP*PM	0.005258	**<0.0001**

—*, Indicates not applicable. The bold values indicate statistical significance P <0.05*.

**Table 3.2:** Multivariate linear mixed-effects regression analysis of the influence of covariates on voriconazole dose required to achieve therapeutic range (mg/kg).

**Factor**	**Coefficient ±SE**	***P*-value**
Intercept	27.040 ± 1.423	**<0.0001**
Age	−0.728 ± 0.133	**<0.0001**
**Phenotypes**		
NM	—	—
IM	−4.468 ± 1.184	**0.0002**
PM	−9.429 ± 1.762	**<0.0001**
**Presence of immunosuppressants**		
No	—	—
Yes	2.266 ± 0.629	**0.0004**
Logarithmic of CRP	−0.515 ± 0.190	**<0.0001**
**Interactive effects of log(CRP) and Phenotypes**		
CRP*NM	—	—
CRP*IM	−0.001 ± 0.255	0.9956
CRP*PM	1.102 ± 0.340	**0.0013**

—*, Indicates not applicable; SE, standard error. The bold values indicate statistical significance P <0.05*.

### Influence of CRP Varied by *CYP2C19* Phenotypes on Voriconazole Concentrations and Dose Requirements

**Figure 3:**
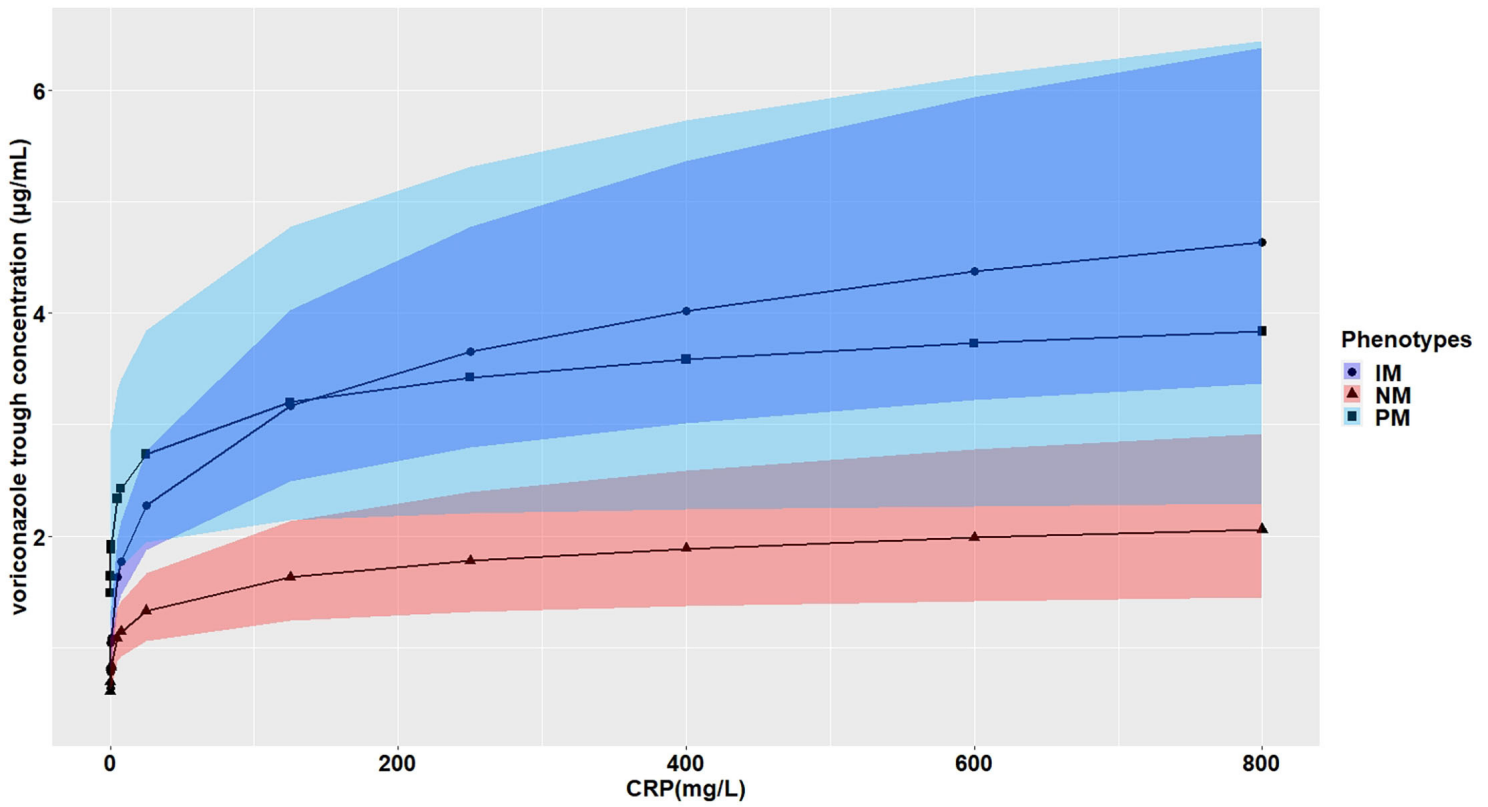
Effects of the CRP levels on the voriconazole trough concentration (mg/ml) according to the *CYP2C19* phenotypes. The figure illustrates the predicted voriconazole CDR as a function of CRP level from the multivariate generalized linear mixed-effects model. The curves with closed triangle, closed circle, and closed square represent children with normal metabolizer (NM), intermediate metabolizer (IM), and poor metabolizer (PM), respectively.

**Figure 4:**
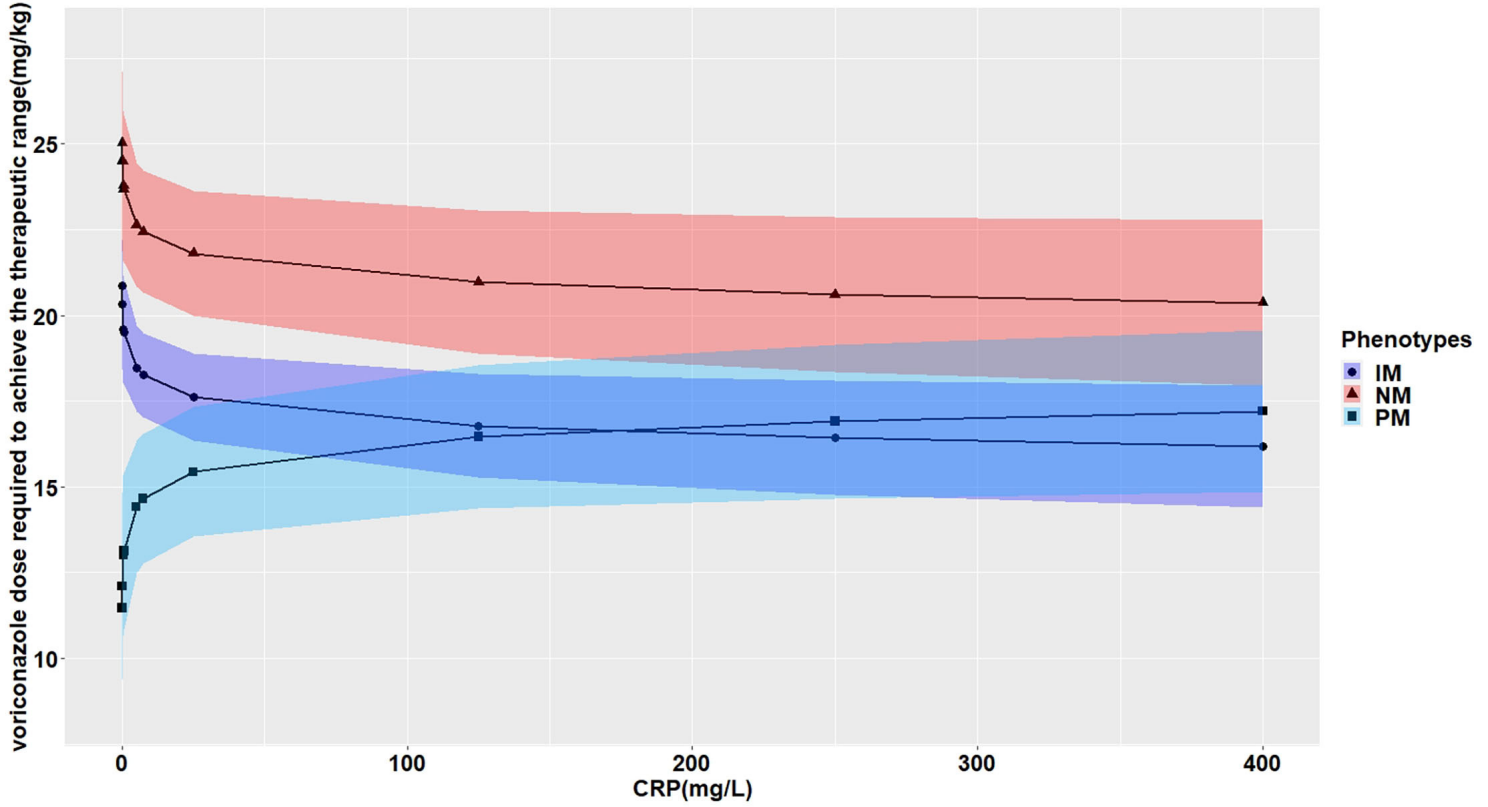
Effects of the CRP levels on the voriconazole dose (mg/kg) required to achieve the therapeutic range according to the *CYP2C19* phenotypes. The figure illustrates the predicted voriconazole dose required to achieve the therapeutic range as a function of CRP level from the multivariate generalized linear mixed-effects model. The curves with closed triangle, closed circle, and closed square represent children with normal metabolizer (NM), intermediate metabolizer (IM), and poor metabolizer (PM), respectively.

The effects of CRP on voriconazole concentrations and dose requirements were identified. The interactive effects of CRP and *CYP2C19* phenotypes on voriconazole exposure and dose requirements were statistically significant in the multivariate linear mixed-effects regression analysis (Tables 3.1, 3.2). Further subgroups analysis confirmed the positive association between CRP level and voriconazole exposure in the *CYP2C19* NM group (*p* < 0.0001), IM group (*p* < 0.0001), and PM group (*p* = 0.0026). Moreover, the impact of CRP on voriconazole concentration was slightly greater in the IM group (coefficient of 0.2605) than in the NM (coefficient of 0.2400) group and PM group (coefficient of 0.2307) (Table 4.1). Figure 3 shows that with an increase of the CRP concentration from 5 to 25, 125, 250, or 400 mg/L, the voriconazole trough concentration was expected to increase from 1.08 to 1.32, 1.62, 1.78, or 1.89 μg/ml, respectively, in the NM group, to increase from 1.63 to 2.27, 3.17, 3.65, or 4.02 μg/ml, respectively, in the IM group, and to increase from 2.33 to 2.73, 3.20, 3.42, or 3.58 μg/ml, respectively, in the PM group. These meant that increases of 20, 120, 245, and 395 mg/L from 5 mg/L in CRP levels were associated with increases in voriconazole trough concentration by 22.22, 50, 64.81, and 75%, respectively, in the NM group, by 39.26, 94.48, 123.93, and 146.63%, respectively, in the IM group, and by 17.17, 37.34, 46.78, or 53.65%, respectively, in the PM group. In the *CYP2C19* NM, IM, and PM groups, CRP concentrations were always associated with the daily dose of voriconazole required to achieve therapeutic range according to the results from the subgroup analyses (Table 4.2). Figure 4 shows that with an increase of the CRP concentration from 5 to 25, 125, 250, or 400 mg/L, the voriconazole dosage required to achieve therapeutic range is expected to decrease from 22.64 to 21.80, 20.97, 20.61, or 20.37 mg/kg, respectively, in the NM group, to decrease from 18.46 to 17.72, 16.78, 16.42, or 16.18 mg/kg, respectively, in the IM group, and to increase from 14.41 to 15.44, 16.46, 16.91, or 17.21 mg/kg, respectively, in the PM group. These meant that increases of 20, 120, 245, and 395 mg/L from 5 mg/L in CRP levels were associated with increases in voriconazole dose requirements by 7.15, 14.23, 17.35, and 19.43%, respectively, in the PM group; with decreases in voriconazole dose requirements by 3.71, 7.38, 8.97, and 10.03%, respectively, in the NM group; and with decreases by 4, 9.10, 11.05, and 12.35%, respectively, in the IM group.

**Table 4.1:** Effects of CRP concentration on voriconazole CDR (μg/ml per mg/kg) on three subgroups of patients based on phenotypes.

**Factor**	**Subgroup based on phenotypes**					
	**NM**		**IM**		**PM**	
	**Coefficient**	***P*-value**	**Coefficient**	***P*-value**	**Coefficient**	***P*-value**
Age	0.09627	**0.0006**	0.06113	**0.0419**	0.05928	0.3776
**Gender**						
Male	—	—	—	—	—	—
Female	−0.02108	0.9234	−0.1791	0.4033	−0.2857	0.4508
**Presence of immunosuppressants**						
No	—	—	—	—	—	—
Yes	0.1862	0.1622	−0.5287	**0.0040**	−0.1649	—
Logarithmic of weight-corrected dose	0.7695	**0.0011**	0.1635	0.2711	0.2360	0.4330
Logarithmic of CRP	0.2400	**<0.0001**	0.2605	**<0.0001**	0.2307	**0.0026**

—*, Indicates not applicable; The bold values indicate statistical significance P <0.05*.

**Table 4.2:** Effects of CRP concentration on voriconazole dose required to achieve therapeutic range (mg/kg) on three subgroups of patients based on phenotypes.

**Factor**	**Subgroup based on phenotypes**					
	**NM**		**IM**		**PM**	
	**Coefficient ±SE**	***P*-value**	**Coefficient ± SE**	***P*-value**	**Coefficient± SE**	***P*-value**
Intercept	27.939 ± 2.178	**<0.0001**	21.099 **±** 1.556	**<0.0001**	21.244 **±** 3.078	**<0.0001**
Age	−0.865 **±** 0.229	**0.0005**	−0.523 **±** 0.186	**0.0075**	−1.060 **±** 0.305	**0.0043**
**Presence of immunosuppressants**						
No	—	—	—	—	—	—
Yes	2.340 **±** 1.220	0.057	2.558 **±** 0.810	**0.0018**	0.193 **±** 1.755	0.9128
Logarithmic of CRP	−0.518 **±** 0.226	**0.0232**	−0.520 **±** 0.173	**0.0030**	0.639 **±** 0.213	**0.0038**

—*, Indicates not applicable; SE, standard error. The bold values indicate statistical significance P <0.05*.

### Other Factors Affecting Voriconazole Concentrations and Dose Requirements

The effects of other factors including age, gender, route of administration, different drug–drug interactions, and liver and renal function on the pharmacokinetics of voriconazole were analyzed by univariate linear mixed-effects regression analysis in advance (data was not shown), affecting factors with *p* < 0.15 included for further multivariate linear mixed-effects regression analysis. No significant effect was observed of gender on voriconazole concentrations and dose requirements in multivariate analysis, but age and immunosuppressant comedication did have a significant effect (Tables 3.1, 3.2).

## Discussion

In the present study, voriconazole trough concentrations demonstrate high interindividual and intraindividual variability in pediatric patients. The variability of voriconazole exposure and dose requirement could be partially explained by *CYP2C19* genotype, CRP concentration, age, and comedication with immunosuppressant.

Throughout voriconazole treatment, longitudinal and repeated voriconazole TDM was conducted in our institution. Consistent with previous studies (28, 32–34), we observed a wide variability in voriconazole exposure under steady state, with 38.1% of trough concentrations out of therapeutic range. Lempers et al. showed that 24.1% of 485 voriconazole trough concentrations and Boast et al. showed 44.2% of 120 samples would not achieve trough concentration of >1 μg/ml (28, 34). Our study showed that 28.4% of 682 voriconazole trough concentrations are below the therapeutic range. In cases of trough concentration <1 and >5 μg/ml, 56.7% of dose increases and 65.9% of dose decreases result in the optimal therapeutic level of 1-5 μg/ml, respectively. The results demonstrate that TDM-based dose adjustment is sorely needed to optimize drug concentrations.

The study of clinical importance of *CYP2C19* genotype on voriconazole pharmacokinetics in pediatric patients is controversial. It has been proposed that voriconazole exposure in immunocompromised children and adolescents could not be predicted based on *CYP2C19* genotype (5, 6). In this study, voriconazole exposure was lower in the *CYP2C19* NM group compared with the IM group and the PM group. Our results corroborate previous findings in pediatric patients (15–17, 19). Walsh et al. (15) and Karlsson et al. (16) showed significantly higher elimination capacity in the NM group than in the IM and PM groups in pediatric population pharmacokinetic analysis. Narita et al. (17) reported higher trough plasma concentrations of voriconazole in the PM and IM groups compared with the NM and UM groups. A recent study demonstrated that patients aged no more than 12 years and more than 12 years required doses of 6.53 ± 2.08 and 3.95 ± 0.85 mg/kg of body weight twice daily (*p* = 0.007) in the *CYP2C19* UM or NM group, patients younger than 12 and older than 12 years required doses of 5.75 ± 1.73 and 4.23 ± 0.76 mg/kg twice daily (*p* = 0.019) in the *CYP2C19* PM or IM group for reaching therapeutic concentration (19). In our study, the median daily dose of voriconazole required to achieve therapeutic range also demonstrated an phenotypic dose effect: 20.8 mg/kg (range, 16.2–26.8 mg/kg) for the *CYP2C19* NM group, 18.2 mg/kg (range, 13.3–21.8 mg/kg) for the *CYP2C19* IM group, and 15.2 mg/kg (range, 10.7–19.1 mg/kg) for the *CYP2C19* PM group, respectively. The difference in dose requirements between the two studies might be explained by the age difference. The median age was 6.0 years (range, 3.0–9.0 years) and only five patients were older than 12 years in our study. As the voriconazole daily dose required varied widely within each phenotype and overlapped considerably across *CYP2C19* phenotypes, we failed to provide dose regimens based on *CYP2C19* phenotypes, whereas our results demonstrated that the recommended doses in voriconazole product characteristics for pediatric patients were very likely not enough for adequate therapeutic drug levels in the *CYP2C19* NM and IM groups in patients younger than 12 years.

Severe infections and inflammation reactions are commonly seen in hospitalized pediatric patients, particularly in immunocompromised pediatric patients. A prospective study reported that inflammation severely affects midazolam clearance in pediatric patients (35). In *in vitro* studies, it was demonstrated that proinflammatory cytokines, such as interleukin 2 (IL-2), IL-6, and tumor necrosis factor α, may negatively regulate the CYP2C19 enzyme (36). Moreover, it has been reported that cytokines may stimulate the production of acute-phase proteins, such as CRP (37). Only two previous studies in pediatric patients showed that CRP levels were significantly associated with voriconazole pharmacokinetics in children 12 years or older, and no effect of inflammation on voriconazole concentration was observed in younger children (24, 25). The CRP concentrations had a significant impact on voriconazole exposure and dose requirement in our study. Moreover, the impact of CRP on the trough concentration of voriconazole was modulated by *CYP2C19* phenotypes, and the extent of increase in voriconazole trough concentration was larger for the IM group than the PM group and the NM group (Table 4.1; Figure 3). Increases of 20, 120, 245, and 395 mg/L from 5 mg/L in CRP levels were associated with an increase in voriconazole trough concentration by 22.22, 50, 64.81, and 75%; by 39.26, 94.48, 123.93, and 146.63%; and by 17.17, 37.34, 46.78, and 53.65% in the NM, IM, and PM groups, respectively. The result was consistent with a previous study in adults (38), whereas a recent meta-analysis demonstrated a smaller effect of inflammation for adult patients with decreased metabolic capacity for CYP2C19 (IM and PM) than those with normal (NM) or elevated metabolic capacity (RM and UM) (39). The study by Gautier-Veyret et al. (40) was inconsistent with ours. In their study, CRP levels tended to positively influence voriconazole concentration in adults with an increased metabolic activity, but had no impact on adults with normal metabolic activity. The conflicting results could be explained by the genetic factors that integrated both *CYP2C19* and *CYP3A* polymorphisms in the latter study. In our study, we also presented the interactive effect between CRP and *CYP2C19* phenotypes on daily dose of voriconazole required to achieve therapeutic range. The dosages required were decreased in the NM group and the IM group by different degrees with the same increase in the CRP level, but increased in the PM group with the same increase in the CRP level (Table 4.2; Figure 4). This may be explained by the phenomenon of inflammation-induced phenoconversion of polymorphic drug-metabolizing enzymes (36).

Age was an influencing factor contributing to the interindividual variability in voriconazole exposures in our population. Hicks et al. (13) reported a correlation between age and higher voriconazole through concentrations corrected for daily dose in pediatric patients, and age also did have a significant impact in a multivariate analysis. *In vitro* and *in vivo* studies demonstrated that voriconazole appears to be more rapid metabolized in pediatric patients compared with that in adults; the maintenance doses/weights needed in pediatric patients were higher (41, 42). Mechanistically, age is closely related to the activity of liver microsomes, the body fat, and total body water, which potentially leads to the difference of metabolism or distribution of voriconazole.

Voriconazole is often treated in combination with immunosuppressant drugs in patients after HSCT. Immunosuppressant therapies, including cyclosporine, tacrolimus, and sirolimus, are substrates of CYP3A4 enzyme. The drug–drug interactions have high clinical relevance because of the inhibition effect of voriconazole on CYP3A4 enzyme, whereas the effect of immunosuppressants on voriconazole exposure is rarely reported. In our study, immunosuppressant treatments were statistically significant covariates explaining interindividual variability in voriconazole exposure and dose requirement in multivariate linear mixed-effects regression analysis. Zeng et al. (43) reported that the cyclosporine concentration was an independent factor of voriconazole concentration variation, but combination of cyclosporine did not affect voriconazole concentration. Hashemizadeh et al. (44) showed no significant correlation between immunosuppressant treatments and voriconazole concentration. Cyclosporine administration was significantly associated with an increase in voriconazole exposure in the plasma, cerebrospinal fluid, and brain in an animal model of cerebral scedosporiosis (45). An *in vitro* study demonstrated that low concentrations of voriconazole was mainly (~93%) metabolized by CYP2C19, whereas CYP3A4 was mainly (~73%) responsible for metabolism in the case of high concentrations of voriconazole (46). As cyclosporine, tacrolimus, and sirolimus are substrates of CYP3A4, voriconazole disposition at high concentration might be modified by competition at the CYP3A4 catalytic site, whereas more *in vitro* and *in vivo* studies are still needed for the conflicting issue about the effect of immunosuppressants on voriconazole exposure.

This study has several limitations. First, this is a retrospective observation study in a single tertiary center. Second, genetic polymorphisms of other proteins (CYP3A4, CYP3A5, FMO) that may influence voriconazole pharmacokinetics were not analyzed. Further prospective studies are still needed to validate our findings and to explore the effect of other possible covariates not included in the present study.

## Conclusion

In this study, we found that influencing factors such as *CYP2C19* phenotype, CRP concentration, age, and presence of immunosuppressants were significantly associated with voriconazole trough concentrations and dose requirements to reach therapeutic range in Chinese pediatric patients. The combination of influencing factors with routine TDM may help to individualize voriconazole dosing and improve clinical outcomes.

## Data Availability Statement

The original contributions presented in the study are included in the article/Supplementary Material, further inquiries can be directed to the corresponding author/s.

## Ethics Statement

The studies involving human participants were reviewed and approved by Nanfang Hospital of Southern Medical University. Written informed consent to participate in this study was provided by the participants' legal guardian/next of kin.

## Author Contributions

JC, YW, and SL performed and designed the research. JC and YW analyzed the data and wrote the article. JC, YH, XF, and YR collected the children's data. All authors contributed to the article and approved the submitted version.

## Funding

This work was supported by the National Natural Science Foundation of China (81703322).

## Conflict of Interest

The authors declare that the research was conducted in the absence of any commercial or financial relationships that could be construed as a potential conflict of interest.

## Publisher's Note

All claims expressed in this article are solely those of the authors and do not necessarily represent those of their affiliated organizations, or those of the publisher, the editors and the reviewers. Any product that may be evaluated in this article, or claim that may be made by its manufacturer, is not guaranteed or endorsed by the publisher.
