# Supplementary figures and images for: Combined Effect of CYP2C19 Genetic Polymorphisms and C-Reactive Protein on Voriconazole Exposure and Dosing in Immunocompromised Children
Source: Front Pediatr. 2022 Mar 21;10:846411. doi: 10.3389/fped.2022.846411 (PMC8978631; doi:10.3389/fped.2022.846411)

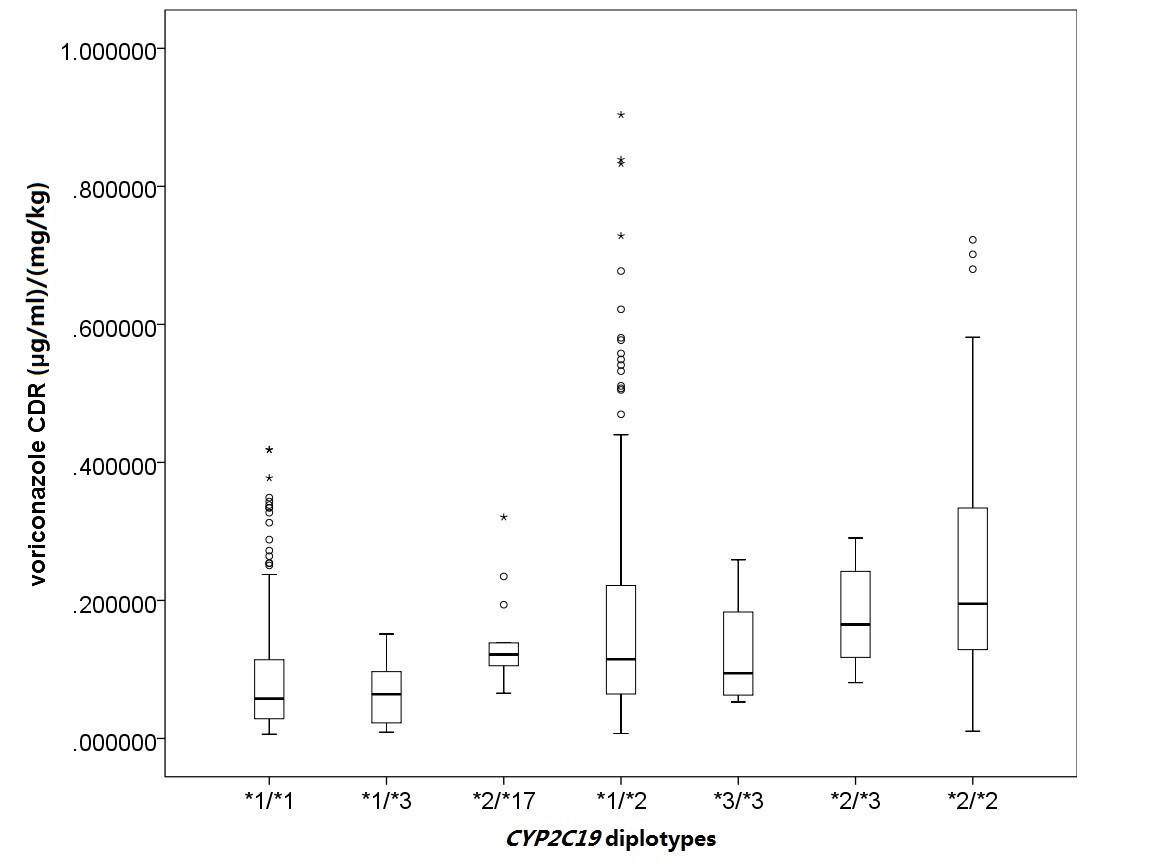

Supplement: Supplementary file 1 [file Image_1.JPEG]

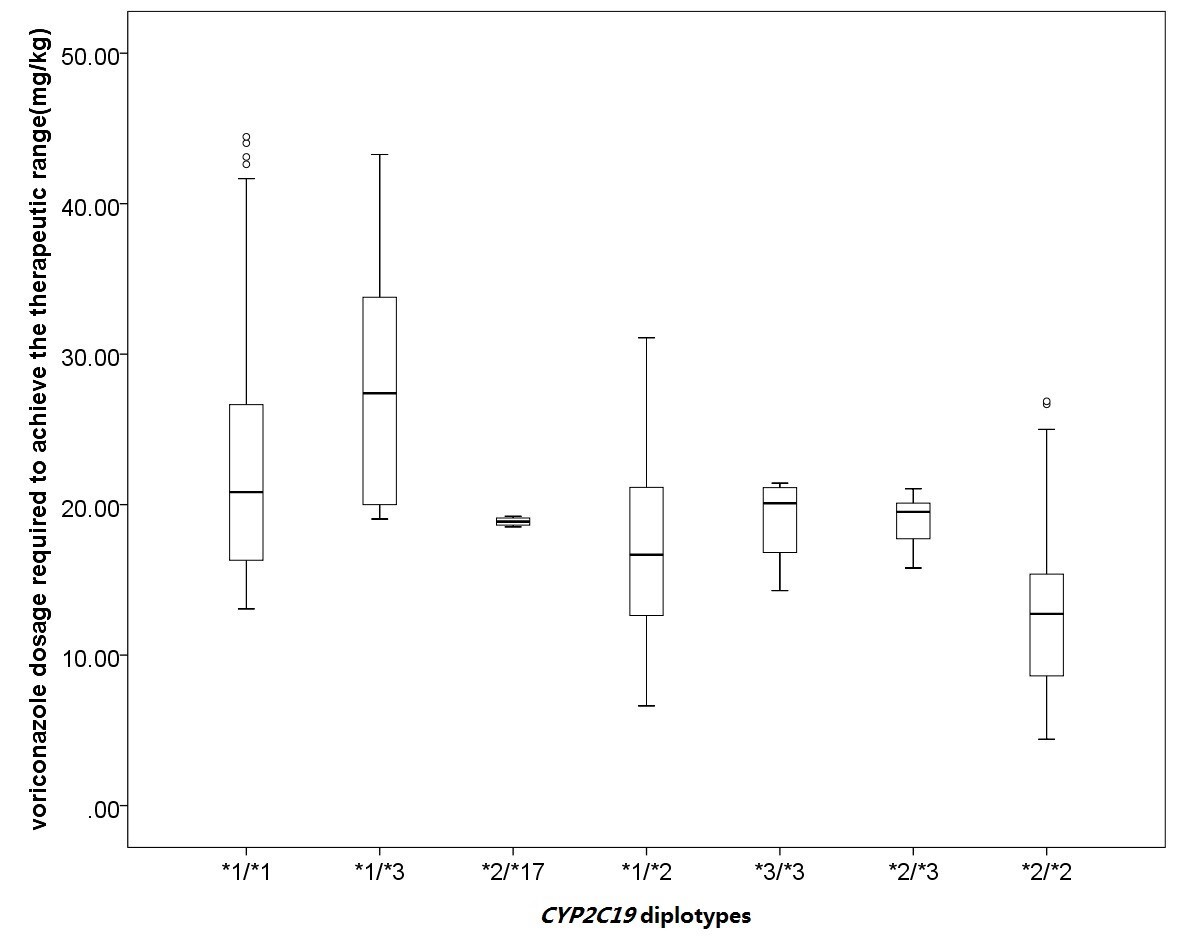

Supplement: Supplementary file 2 [file Image_2.JPEG]
